# Effects of E-Cigarette Exposure on Prenatal Life and Childhood Respiratory Health: A Review of Current Evidence

**DOI:** 10.3389/fped.2021.711573

**Published:** 2021-08-20

**Authors:** Federica Mescolo, Giuliana Ferrante, Stefania La Grutta

**Affiliations:** ^1^Department of Health Promotion, Mother and Child Care, Internal Medicine and Medical Specialties, University of Palermo, Palermo, Italy; ^2^Institute for Biomedical Research and Innovation, National Research Council, Palermo, Italy

**Keywords:** E-Cigarettes, EVALI, parental perception, passive exposure, respiratory health

## Abstract

In the last decade, widespread use of E-cigarettes (EC) has occurred all over the world. Whereas, a large amount of evidence on harm to children from conventional cigarette exposure is available, data on health effects in this population throughout different vulnerability windows are still a matter of concern. Exposure to EC during pregnancy may compromise placental function, resulting in fetal structural abnormalities. Specifically, this may cause physio-pathologic changes in the developing lung, which in turn may impair respiratory health later in life. Furthermore, there is evidence that using EC can cause both short- and long-term respiratory problems in the pediatric population and there is great concern for future young people with nicotine addiction. The low parental perception of the risks connected to EC exposure for children increases their susceptibility to harmful effects from passive vaping. This minireview aims to summarize the current evidence focusing on: (i) prenatal effects of EC passive exposure; (ii) post-natal respiratory effects of EC exposure in youth; (iii) parental attitudes toward EC use and perception of children's health risks connected to EC exposure; and (iv) addressing gaps in our current evidence.

## Introduction

In the last decade, widespread use of E-cigarettes (EC) has occurred all over the world. EC are electronic nicotine delivery systems (ENDS) composed of a battery-powered heating element that aerosolizes a liquid solution comprising nicotine, propylene glycol (PG), and vegetable glycerin (VG) as the main components. PG, VG, glycerol, and a variety of flavors (1) are the principal components of vaping fluids. The heterogeneity of vaping fluid composition results in a large variety of nicotine concentrations in liquids in different brands and in the choices made by users (2).

After the initial models similar to tobacco cigarettes in size and shape, EC manufacturers have developed various devices with refilling reservoirs and different heating power, which allow the user to mix e-liquids and choose the temperature of the vapor (3). However, the possibility of making up e-liquids using variable concentrations of nicotine, other non-nicotinic components, and different flavors, associated with the absence of standardization for EC content by manufacturers, results in inconsistent quality control processes (4).

The use of flavors may have made EC more popular, especially among pregnant women and adolescents (5). The attraction of flavored products for pregnant women is likely due to alterations in taste, cravings, nausea and high sensitivity to bitter tastes which commonly occur during pregnancy. Moreover, flavorings increase the appeal to youths who increasingly have started smoking EC, serving as a gateway to cigarette smoking (5). In addition, the fact that EC functioning is not based on combustion and virtually does not produce a side-stream aerosol has led to their widespread use in homes, cars, and other indoor settings (6). Indeed, parents living in socio-economically disadvantaged circumstances especially tend to consider EC as potentially helpful to protect their children from passive smoke exposure in the home (7).

**Figure 1:**
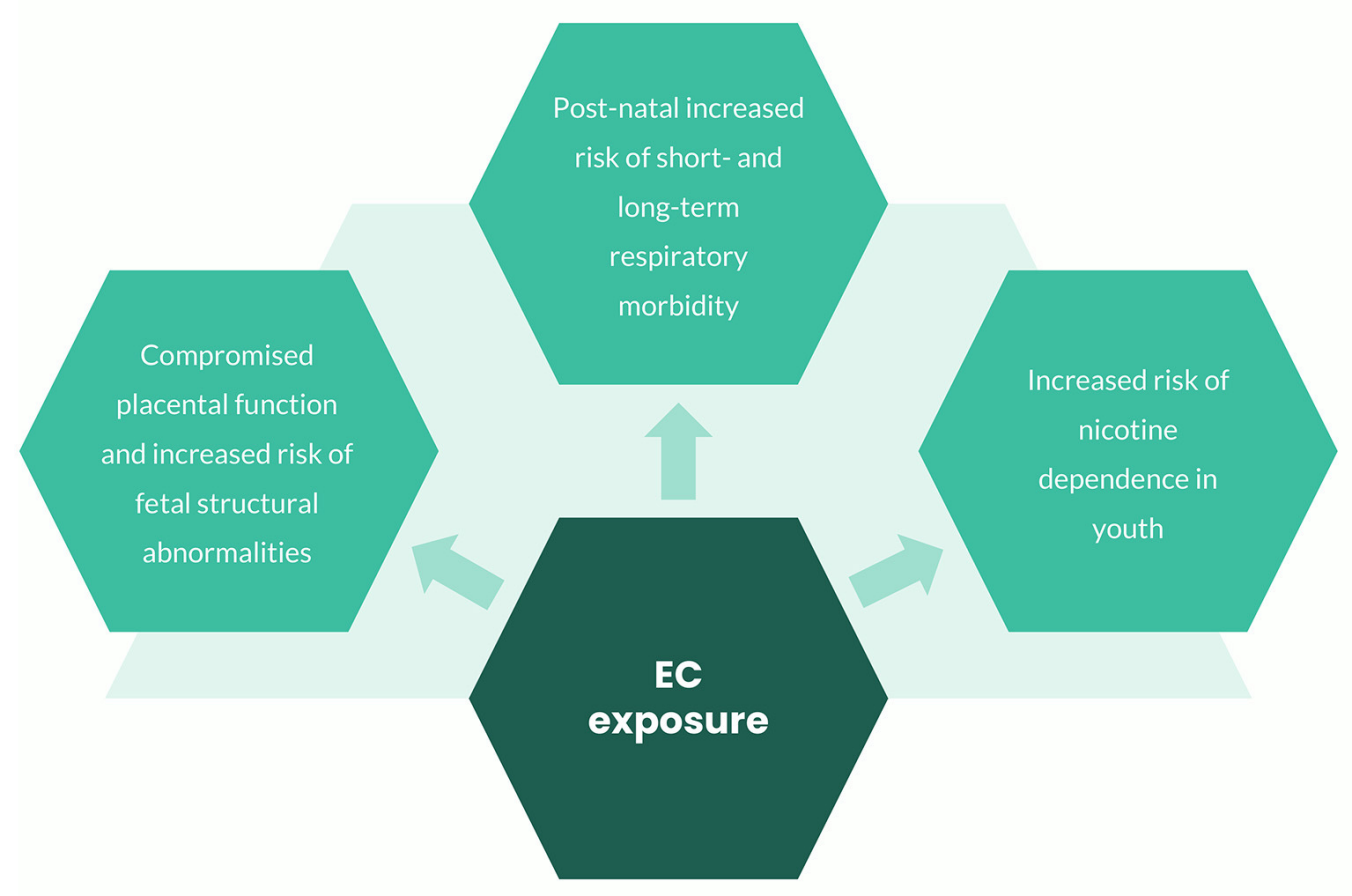
Effects related to EC exposure throughout different vulnerability windows.

To date, a large amount of evidence on harm to children from conventional cigarette exposure is available (8), whilst data on health effects in this population throughout different vulnerability windows remain a matter of concern. There is growing evidence that exposure of pregnant women to EC may impair placental function and may result in fetal structural abnormalities. Furthermore, it appears that using EC can cause both short- and long-term respiratory problems in the pediatric population and there are fears that a future generation of youth may be addicted to nicotine (Figure 1).

This minireview aims to summarize the current evidence focusing on: (i) prenatal effects of EC passive exposure; (ii) post-natal respiratory effects of EC exposure in youth; (iii) parental attitudes toward EC use and perception of children's health risks connected to EC exposure; and (iv) addressing gaps in our current evidence.

## Prenatal Effects of EC Passive Exposure

The majority of negative effects of EC exposure in prenatal life have been studied using *in vitro* and animal models, with limited data obtained from studies conducted in humans. Emerging evidence suggests that prenatal EC exposure may determine placental compromise and consequent dangerous effects for the fetus. Nicotine is known to be responsible for most of the effects, but vaping aerosol contains many harmful chemicals other than nicotine. Nicotine has a para-sympathomimetic stimulant effect. Its ability to cross the placental barrier is responsible for the high concentration in fetal serum and amniotic fluid (9).

The risks of smoking EC during pregnancy are still largely unknown. However, evidence suggests that even nicotine-free EC aerosols may cause harm to the fetus. The HTR8/SVneo cells derived from transfected cells of human chorionic villi have been used to study the function of placental cells exposed to flavorless EC without nicotine, showing a significant reduction in trophoblast impairment and angiogenesis functions, which are vital for placental circulation (10). These results suggest that placental cells may be vulnerable to exposure to EC aerosols, even in the absence of nicotine, but these findings require further studies.

A negative effect of EC has been demonstrated in cardiac differentiation of human embryonic stem cells *in vitro* (11). Moreover, when gravid mice were exposed to e-vapors containing nicotine, there was an increase in pro-inflammatory cytokines, namely TNF-a, in the lungs of their offspring, whereas IL-1b was suppressed. This occurred both with nicotine-free e-vapors and nicotine-containing ones; therefore, these effects are probably not explained by nicotine but rather by by-products (12). Indeed, e-vapors also contains toxins along with heavy metals and carcinogenic nitrosamines. Moreover, compounds used for flavoring can themselves be toxic or break down into compounds which may cause toxicity, inflammation, and oxidative stress in the pregnant woman and can built up in the fetus, with consequences for intrauterine development. Murine models have also demonstrated that maternal e-vapor exposure during pregnancy can affect respiratory and neurological functions (13). With regard to neurodevelopmental consequences of maternal EC use, adverse behavioral outcomes have been observed in mice adult offspring. Even without nicotine, being exposed daily to PG/VG all through the gestation period may disrupt learning and memory performance and cause a rise in neuro-inflammation (14). Previously, Nguyen et al. identified changes in the behavior of the offspring of mice exposed to EC vapor with and without nicotine during pregnancy: mice showed a higher rate of short-term memory deficits, learning disabilities, and increased anxious behavior, were more hyperactive and tended to explore the environment in risky ways compared to non-exposed ones. In addition, global DNA methylation was increased and it was found that 13 key genes were identified to be significantly altered in the brains of EC exposed offspring mice compared to the non-exposed ones (15). A risk of renal impairment, including increased kidney markers of oxidative stress, renal inflammation, and fibrosis along with reduction of kidney volumes and function in the adult offspring was associated with EC use in pregnancy, and these effects were found to be independent of the presence of nicotine in the aerosols (16).

Harmful effects due to EC exposure in pregnancy have also been reported in humans. Cardenas et al. evaluated tobacco use in a cohort of 248 pregnant women, finding that those who used ENDS had significantly lower gestational age-specific birth weights than ones that did not smoke. The relative risk (RR) of small-for-gestational-age (SGA) newborns was five times higher than for women that did not smoke at all (17). Clemens et al. compared birth outcomes among 76 women who smoked both EC and tobacco cigarettes and women who did not smoke at all. They found that women who smoked both EC and tobacco cigarettes had a 7.8 times higher risk for SGA newborns than women that did not smoke at all (18). However, it should be pointed out that in both the studies population was limited in size; therefore, findings may not be generalizable. Moreover, most of the enrolled women were dual users and analyses were not adjusted for cotinine level in order to evaluate differential impact of tobacco exposure vs. nicotine exposure on SGA. Consequently, the reported results should be interpreted with caution, given that tobacco smoking is associated with SGA. In a more recent survey on 1,594 pregnant women, most of them dual users, estimated adjusted RR for SGA for dual users was slightly higher with respect to cigarette-only smokers [1.8 (95% CI: 1.0, 3.4) vs. 1.7 (95% CI: 1.1, 2.7)]. These RR estimates increased after correcting for tobacco use misclassification. Dual users who continued using ENDS but stopped smoking cigarettes had an increased risk for SGA compared with non-tobacco users [3.2 (95% CI: 1.5, 6.6)] (19). Furthermore, data from 55,251 pregnant women who participated in the Phase 8 survey of the Pregnancy Risk Assessment Monitoring System between 2016 and 2018 confirmed that EC use cannot be considered a safer alternative to conventional cigarette (CC) smoking during pregnancy, as EC and CC users showed not significantly different risk on SGA, low birth weight and preterm birth (20). Lastly, with regard to the effects of the chemical components of e-vapors, they have not been thoroughly defined in human studies so far, but it has been suggested that certain populations, including pregnant women, could process PG differently to the general population, being prone to PG accumulation (21).

Overall, both in animal and human studies evidence of harmful effects related to EC exposure during prenatal life has been provided.

## Post-Natal Respiratory Effects of EC Exposure in Childhood

To date, little information is available regarding the effect of EC exposure on children's respiratory health. The available data on pulmonary symptoms are inconsistent. A survey conducted on 3,488 schoolchildren aged 6–17 years in Switzerland investigated the association between active smoking of CC, EC and shishas and current respiratory symptoms, and found that dyspnea and wheeze were more widespread among frequent smokers, i.e., those who smoked at least once/week (30 and 12%, respectively), and occasional smokers (22 and 13%) than in never smokers [19 and 8%, *p* < 0.05; (22)]. Similarly, a survey conducted on 44,662 Chinese students (mean age 14.6 years), reported that EC use was significantly associated with respiratory symptoms, e.g., cough or phlegm for 3 consecutive months in the past 12 months [adjusted OR (AOR), 1.28; 95% CI, 1.06–1.56)] (23). Likewise, in a recent study on 2,086 adolescents aged 16–18 years who took part in the Southern California Children's Health Study, self-reported wheeze in the last 12 months was associated with current (OR [95% CI] = 1.86 [1.28–2.71]) but not past use of EC. In the same study, chronic bronchitis symptoms during the previous year were associated with both past (OR, 1.85; 95% CI, 1.37–2.49) and present use of EC (OR [95% CI] = 2.02 [1.42–2.88]). The risk of bronchitis symptoms was directly proportional to the number of days of EC use in the past month (OR [95% CI] = 1.66 [1.02–2.68] for 1–2 days and OR [95% CI] = 2.52 [1.56–4.08] for >3 days), compared with non-EC users (*p* for trend = 0.001) (24). More recently, in a cohort study of 7,049 adolescents, the association of EC use alone with wheezing in the past 12 months proved not to be significant (25). Overall, it should be pointed out that data from the aforementioned studies are self-reported and may be subjected to recall bias. Moreover, symptoms may have been interpreted in an inaccurate way, especially by young children. Therefore, higher standards for future studies are essential in order to obtain more robust results in this field of research.

By contrast, with regard to associations between EC use, especially when combined with smoking of other products, and asthma risk in adolescents, more evidence has been reported. A cross-sectional study investigating the effects of active, passive, and EC smoking in Korean adolescents, including 4,890 with asthma in the last 12 months, reported significant associations between active and passive smoking and asthma (respectively: adjusted (A) OR [95% CI] of smoking ≥20 days/month = 1.57 [1.38–1.77], *p* < 0.001; AOR [95% CI] of smoking ≥5 days/week = 1.40 [1.28–1.53], *p* < 0.001). In addition, the authors found that asthma was significantly more frequent in the EC group (AOR [95% CI] = 1.13 [1.01–1.26], *p* = 0.027). Nonetheless, EC smoking in the last month proved not to be significantly associated with lifetime asthma after adjusting for active and passive smoking, which thus appeared to be more influential in previous asthma history than recent EC smoking (26). In another cross-sectional study on a large sample of 35,904 high school students (mean age 16.4 years) in South Korea, the authors found that EC users had an increased association with asthma diagnosis (AOR [95% CI] = 2.74 [1.30–5.78], *p* < 0.01) in comparison with non-EC users, suggesting that EC use may be a risk factor for asthma. Of note, current EC users had the highest risk for severe asthma, defined as the number of days (≥4) absent from school in the past year due to asthma symptoms, compared to non-EC users [AOR [95% CI] = 15.42 [5.11–46.57], *p* < 0.001; (27)]. Similarly, in a survey on 6089 adolescents (mean age 15.8 years) in Hawaii, the authors reported that current use of EC showed a significant association with current asthma (AOR [95% CI] = 1.48 [1.26–1.74]) and a marginal one with asthma at any time (AOR [95% CI] = 1.22 [1.07–1.40]) (28). A recent study on 21,532 U.S. adolescents (age range: 14–18 years), in which use of an e-vapor product was associated with asthma, showed an additional harmful effect on asthma of the combined use of vapor products with marijuana and cigarette smoking: use of an e-vapor product was associated with asthma, and this association was even more evident when its use was coupled with marijuana, particularly when cigarette smoking was also involved. When adjusting for frequent cigarette smoking and marijuana use, frequent use of an electronic vapor product (≥10 days/month) was significantly associated with asthma [AOR [95% CI] = 1.31 [1.11–1.54], *p* < 0.01; (29)]. Overall, it should be taken into account that the lack of cross-validation by physician diagnosis of asthma may limit these findings. Because all the aforementioned studies were based on self-reported questionnaires, undiagnosed, or misclassified asthma was likely. Hence, the reported findings should be corroborated with medical records, clinical assessment measures, or examination by physicians, and this should be considered for interpretation of the results.

Asthma exacerbations were found to be associated to both active and passive EC smoking. Data from the 2012 Florida Youth Tobacco Survey, involving 36,085 high school students, reported that prevalence of ever having used EC and having used them in the past 30 days among those who reported asthma was 10.4 and 5.3% respectively, and this was significantly higher than in those without asthma (ever use = 7.2% and past 30-day use = 2.5%; *p* < 0.01). Interestingly, among students with asthma, use of EC in the past 30 days was positively associated with an asthma attack in the past 12 months (AOR [95% CI] = 1.78 [1.20–2.64]) (30). Similarly, the 2016 Florida Youth Tobacco survey showed that among students with a self-reported diagnosis of asthma (*n* = 11,830), secondhand ENDS aerosol exposure was associated with higher risk of asthma attacks in the past 12 months (AOR [95% CI] = 1.27 [1.11–1.47]) (31). It should be acknowledged that both these studies did not provide details about the variable “asthma attack/exacerbation” and this should be considered when interpreting the reported findings.

In the last few years EC use has been associated with an emergent condition known as “EC vaping associated lung injury” (EVALI). A retrospective case series of six patients with a median age of 17 years with confirmed or probable EVALI and admitted to pediatric intensive care described the implications of vaping on respiratory morbidity among adolescents, raising the concern that patients with EVALI may have lasting lung impairment (32). Indeed, it has been previously demonstrated that a history of respiratory illness and/or a respiratory diagnosis in childhood identified a group of cigarette smokers particularly vulnerable to the harmful effects of smoking (33). Such effect has not been reported so far with regard to EC use in youth. In the study by Reddy et al., half of the patients had a pre-existing diagnosis of asthma, two were positive for Rhinovirus infection, and four had mental health comorbidities. In addition, all patients reported both nicotine and THC use. Though the clinical and radiological findings were similar to those described in confirmed cases of adult EVALI, the study cohort was too small to draw firm conclusions about an association with EC use. Moreover, pulmonary function tests at discharge were compromised only in one patient with pre-existing diagnosis of asthma. Therefore, due to such limitations, these results cannot be generalized and should be prudently evaluated. Indeed, further investigation is required to clarify to what extent EC use contributes to severe acute lung injury in patients smoking multiple agents and whether asthmatics could be more susceptible to potential harmful effects of EC use (32). Overall, though the evidence is limited, it suggests that use of ENDS can cause both short-term respiratory morbidity (i.e., cough, chest pain, shortness of breath) and long-term abnormal spirometry (most commonly obstructive pattern) in the pediatric population (34). According to a very recent systematic review, pediatric EVALI has been found in patients as young as 13 years of age and often included respiratory symptoms. Typical findings were the presence of ground-glass opacities on computed-tomography and leukocytosis. Treatment mainly involved the use of corticosteroids, antibiotics, and ventilatory support, and extracorporeal membrane oxygenation, both when respiratory failure occurred; outcomes ranged from complete or near complete recovery of lung function to death (35). With regard to lung function, in 15 adolescents hospitalized with EVALI, Carroll et al. found that, before discharge, just over half of patients had abnormal spirometry values with a restrictive pattern being twice as frequent as obstruction. Sixty percent of those who underwent diffusion studies showed decreased capacity to diffuse carbon monoxide (DLCO). About 4.5 weeks after discharge in the majority of patients' spirometry continued to be abnormal (most frequently an obstructive pattern) (36). In addition, in 13 hospitalized adolescents with EVALI, Rao et al. showed that corticosteroids produced an improvement in spirometry and DLCO (37). Detection of toxicants in bronchoalveolar lavage (BAL) fluid from patients with EVALI can provide direct information on exposure within the lung. Recently, data from Blount et al. linking the offending agent in EC showed that Vitamin E acetate was associated with EVALI in a convenience sample of 51 patients including adolescents aged 16 years. The findings of the concentrations of vitamin E acetate in BAL fluid from EVALI patients and the absence of vitamin E acetate in BAL from healthy controls, and also the absence of other toxicants in nearly all BAL fluid samples from EVALI patients suggest that Vitamin E acetate may play a role in EVALI. Further studies are requested to support a cause-effect relationship between exposure to vitamin E acetate and the lung injury observed in patients with EVALI (38). Similarly, a recent study on adults found vitamin E acetate in all BAL specimens of a convenience sample of 29 EVALI patients; however, more studies are needed, since it is possible that more than one compound in EC could contribute to lung injury, and evidence is still not robust to rule out contribution of other toxicants to EVALI (39). Another study linked offending agent in EC to EVALI, in contrast to other types of smoking. In particular, Navon et al. reported that among adults reporting use of THC-containing EC or vaping products, EVALI patients had higher risk of reporting exclusive and frequent use of THC-containing products which were mainly obtained from informal sources. This reinforces current recommendations not to use EC or vaping products containing THC, especially when acquired from informal sources such as off the street, from a dealer, or from a friend (40).

Overall, the available evidence suggests that both active and passive EC smoking put children's and adolescents' respiratory health at risk (Table 1). This is a real concern mainly for those who start smoking EC early in life. Moreover, the long-term impact of EC smoking on respiratory symptoms and pulmonary function needs to be further elucidated, especially in patients with EC-associated lung injury.

**Table 1:** Respiratory effects of EC exposure in childhood.

**References**	**Country**	**Study type**	**Study population**	**Aim and study procedures**	**Results**	**Comments**
**EC EXPOSURE AND RESPIRATORY SYMPTOMS**						
Mozun et al. (22)	Switzerland	School-based cross-sectional study conducted in 2013–2016	3,488 children aged 6–17 years	Investigating the association between active smoking of conventional cigarettes, EC and shishas and current respiratory symptoms by validated questionnaire	Dyspnea and wheeze were more frequent among frequent smokers, i.e., those who smoked at least once/week (30 and 12%, respectively), and occasional smokers (22 and 13%) than in never smokers (19 and 8%, *p* < 0.05)	Smoking EC and any other product, even when occasional, was associated with respiratory symptoms
Wang et al. (23)	China	Cross-sectional school-based study conducted between 2012 and 2013	44,662 students (mean age 14.6 years)	Investigating the association between active smoking of EC and current respiratory symptoms by questionnaire	EC use was significantly associated with respiratory symptoms, e.g., cough or phlegm, for 3 consecutive months in the last 12 months (AOR, 1.28; 95% CI, 1.06–1.56)	EC use may independently predict respiratory symptoms
McConnell et al. (24)	U.S.	Cross-sectional school-based study conducted in 2014	2,086 adolescents aged 16–18 years	Investigating the associations of EC use with chronic bronchitis symptoms and wheeze by questionnaire	Self-reported wheeze in the last 12 months was associated with current (OR [95% CI] = 1.86 [1.28–2.71]) but not with past use of EC. Chronic bronchitis symptoms during the previous year were associated with both past (OR, 1.85; 95% CI, 1.37–2.49) and current use of EC (OR [95% CI] = 2.02 [1.42–2.88]). The risk of bronchitis symptoms increased with the number of days of EC use in the last month (OR [95% CI] = 1.66 [1.02–2.68] for 1–2 days) and (OR [95% CI] = 2.52 [1.56–4.08] for >3 days), compared with EC never-users (*p* for trend = 0.001)	Adolescent EC users had increased rates of chronic bronchitis symptoms
Tackett et al. (25)	U.S.	Cohort study conducted between 2015 and 2018	7,049 adolescents aged 12–17 years	Examining the association between EC use and self-reported wheezing by questionnaire	There was no significant association between EC use alone and wheezing in the past 12 months was not significant (AOR for EC use in the past year, 1.37 [95% CI, 0.91–2.05]; in the past 30 days, 1.35 [95% CI, 0.63–2.88]; in the past 7 days, 0.74 [95% CI, 0.28–1.97]; *p* = 0.33)	EC use alone was not associated with increased risk of wheezing
**EC EXPOSURE AND ASTHMA**						
Kim et al. (26)	South Korea	Cross-sectional web-based survey conducted between 2011 and 2013	216,056 participants (4,890 asthmatics) aged 12–18	Investigating the associations of active, passive, and EC smoking with asthma	Significant associations between both active and passive smoking with asthma (respectively: AOR [95% CI] of smoking ≥20 days/month = 1.57 [1.38–1.77], *p* < 0.001; AOR [95% CI] of smoking ≥5 days/week = 1.40 [1.28–1.53], *p* < 0.001). Asthma prevalence was significantly higher in the EC group (AOR [95% CI] = 1.13 [1.01–1.26], *p* = 0.027)	There was no significant association between EC smoking in the last month and was not significantly associated with lifetime asthma after adjusting for active and passive smoking, which were thus considered to have a bigger role in previous asthma history than recent EC smoking
Cho and Paik (27)	South Korea	Cross-sectional web-based survey conducted in 2014	35,904 high school students (mean age 16.4 years)	Investigating the association between EC use and asthma	EC users had an increased association with asthma diagnosis (AOR [95% CI] = 2.74 [1.30–5.78], *p* < 0.01) than EC never-users. Current EC users were more at risk for severe asthma compared to “never EC” users (AOR [95% CI] = 15.42 [5.11–46.57], *p* < 0.001)	Use of EC may be a risk factor for asthma
Schweitzer et al. (28)	U.S.	Cross-sectional school-based survey conducted in 2015	6,089 adolescents (mean age 15.8 years)	Investigating the association between EC use and asthma by means of a questionnaire	There was a significant association between current EC use and current asthma (AOR [95% CI] = 1.48 [1.26–1.74]) and marginally associated with any asthma ever (AOR [95% CI] = 1.22 [1.07–1.40])	EC use is an independent risk factor for current asthma
Han et al. (29)	U.S.	Cross-sectional surveys conducted in 2015 and in 2017	21,532 adolescents (age range: 14–18 years)	Investigating the association between EC use and asthma by questionnaire	Use of an e-vapor product was associated with asthma especially when coupled with marijuana, and even more when also combined with cigarette smoking (AOR [95% CI] = 1.74 [1.44–2.10], *p* < 0.01). When adjusting for frequent cigarette smoking and marijuana use, there was a significant association between frequent use of an e-vapor product (≥10 days/month) and asthma (AOR [95% CI] = 1.31 [1.11–1.54], *p* < 0.01)	E-vapor product use has additional harmful effects on asthma when combined with marijuana and cigarette smoking
**EC EXPOSURE AND ASTHMA ATTACKS**						
Choi and Bernat (30)	U.S.	Cross-sectional survey conducted in 2012	36,085 middle- and high-school students (mean age 16.08 years)	Investigating the association between EC use and asthma attacks by questionnaire	The prevalence of ever and past-30-day use of EC among those who reported asthma was 10.4 and 5.3%, respectively, which was significantly higher than in those without asthma (ever use = 7.2% and last 30-day use = 2.5%; *p* < 0.01). Among students with asthma, past-30-day use of EC was positively associated with reporting an asthma attack in the past 12 months (AOR [95% CI] = 1.78 [1.20–2.64])	EC use may have adverse health effects among youth, especially those with asthma
Bayly et al. (31)	U.S.	Cross-sectional survey conducted in 2016	11,830 students with asthma (age range: 11–17 years)	Investigating the association between EC use and asthma exacerbations by means of questionnaire	Secondhand ENDS aerosol exposure was associated with higher risk of asthma attacks in the last 12 months (AOR [95% CI] = 1.27 [1.11–1.47])	Secondhand exposure to ENDS aerosols may be related to asthma symptoms in youth
**EC EXPOSURE AND EVALI**						
Reddy et al. (32)	U.S.	Single-center retrospective case series	6 adolescents (median age: 17 years)	To characterize EVALI in critically ill adolescents	Broad spectrum of clinical presentation, testing and imaging	There is increasing concern that patients with EVALI will have lasting lung impairment
Kaslow et al. (34)	U.S.	Single-center prospective case series	7 adolescents (age range: 15–17 years)	To characterize EVALI in adolescents	Broad spectrum of clinical presentation, testing, and imaging	ENDS use can cause respiratory damage in the pediatric population in both the short and the long term
Carroll et al. (36)	U.S.	Single-center prospective case series	15 adolescents (mean age: 17.1 years)	To describe short-term pulmonary function abnormalities in adolescents with EVALI	Impairment of pulmonary function tests	Serial testing of pulmonary function could be useful in determining whether patients achieve full resolution of symptoms or whether there will be evidence of irreversible pulmonary impairment
Rao et al. (37)	U.S.	Retrospective chart review	13 adolescents (mean age: 15.9 years) hospitalized with EVALI	Describing diagnosis, evaluation, and management of pediatric EVALI	The use of corticosteroids resulted in improvement in spirometry and DLCO; a 6-min walking test was useful for identifying residual lung disease in patients who had been taken off oxygen due to clinical improvement with glucocorticoid treatment	Lung function deficits due to EVALI appear to be reversible with Steroids. The lone patient who required veno-venous extracorporeal membrane oxygenation (VV-ECMO), and tracheostomy was discharged with a home ventilator and weaned 110 days after initial hospital admission

## Biological Evidence of Harmful Effect of EC on the Airways

Despite the lack of standard *in vitro/ex-vivo* and *in vivo* models, data from available studies support the harmful effect of EC on airway biology. In Table 2 the most recent evidence has been summarized.

**Table 2:** Biological evidence of EC on the airways.

**References**	**Aim**	**Study procedures**	**Results**	**Comments**
**EC BIOLOGICAL EFFECTS** ***IN VITRO/EX-VIVO***				
Escobar et al. (41)	To compare mucin response to EC generated aerosols from propylene glycol (PG) and glycerol (GLY) in airway cells from non-smokers (NS) and smokers (S).	Assessment of mucins (MUC5AC and MUC5B) levels in human nasal epithelial cells (hNEC) from NS and S exposed to aerosols from PG, GLY, and PG:GLY mixture at 55:45 (vol/vol) with freebase nicotine or nicotine salt.	The aerosol from the GLY exposure increased MUC5AC levels in hNECs from NS, and induced pro-inflammatory responses in hNECs from S. The PG:GLY with freebase nicotine exposure increased MUC5AC and MUC5B levels in hNECs from NS.	EC generated aerosols from the humectants, mostly GLY, and type of nicotine caused differential effects in airway epithelial cells from NS and S.
Carson et al. (42)	To characterize mucus hypersecretion and accelerated ciliary beat frequency (CBF) subsequent to a single exposure to tobacco smoke (TS) or EC vapor (EV) on cultured human airway epithelium.	Assessment of mucus hypersecretion and CBF at Air-liquid interface (ALI) airway epithelial cultures of non-smoking (NS) human subjects exposed to TS or EV.	Both TS and EV exposures resulted in similar acute patterns of decline of CBF and induced morphologic differences in secretory function.	Similar pattern of epithelial response to acute TS or EV exposure.
Woodall et al. (43)	To investigate whether exposure to PG or PG mixed with vegetable glycerin (VG) (PG/VG) would reduce glucose uptake in human airway epithelial cells.	Exposure of H441 or human bronchiolar epithelial cells (HBECs) to PG and PG/VG.	Inhibited glucose uptake and mitochondrial ATP synthesis in H441 and HBECs exposed to PG and PG/VG. Reduced transepithelial electrical resistance, and compromised glucose transport function in PG/VG exposed cells.	Short-term exposure to PG/VG, decreases glucose transport in airway cells.
Herr et al. (44)	To compare the acute effects of traditional cigarettes (TCIGs) and EC exposure on host defense, inflammation, and cellular activation of cell lines and primary differentiated human airway epithelial cells (pHBE).	*In vitro:* exposure of Calu-3 and NCI-H292 cell lines to TCIG-smoke or EC-vapor. *Ex-vivo: e*xposure of pHBE cells to TCIG-smoke or EC-vapor.	*In vitro:* significant negative effects of TCIGs on host defense and barrier integrity in Calu-3 cells in comparison with EC-exposed cells. Significant increased IL-8 secretion from Calu-3 cells in EC-exposed cells, similar to the amount found after TCIG-exposure. *Ex-vivo:* moderate increase of IL-8 secretion from Calu-3 cells in EC-exposed cells as compared to TCIGs exposed cells.	Overall less effect of EC on epithelial cells in comparison with TCIG
Ween et al. (45)	To assess the effect of EC constituents, 3 E-liquid apple flavors, nicotine, VG, and PG, on bronchial epithelial cell viability, apoptosis, and cytokine secretion and macrophage phagocytosis of apoptotic airway cells and phagocytic recognition molecules.	*In vitro:* exposure of THP-1 and 16HBE cell lines to 3 E-liquid apple flavors, nicotine, VG, and PG. *Ex-vivo:* exposure of pHBE cells to 3 E-liquid apple flavors, nicotine, VG, and PG.	*In vitro:* EC causes decreased macrophage efferocytosis. *Ex-vivo:* EC vapor increases pHBE cells necrosis and apoptosis. Reduced secretion of Tumor Necrosis Factor (TNF)-α, Interleukin (IL)-6, Interferon gamma-induced Protein (IP)-10, Macrophage Inflammatory Protein (MIP)-1α and MIP-1β was observed for all flavor variants.	EC can cause bronchial epithelial apoptosis and macrophage efferocytosis dysfunction, and alter bronchial epithelial cell cytokine secretion pathways in a flavor-dependent manner.
Ween et al. (46)	To investigate the flavoring profile of 10 different flavored E-liquids.	*In vitro:* assessment of bronchial epithelial cell viability and apoptosis, phagocytosis of bacteria and apoptotic cells by macrophages after exposure to EC vapor extract. *Ex-vivo*: validation in normal human bronchial epithelial cells (NHBE) and alveolar macrophages (AM) from healthy donors.	*In vitro*: flavor dependent necrosis/apoptosis in 16HBE cells and THP-1 differentiated macrophages phagocytosis. Banana and Chocolate reduced surface expression of phagocytic target recognition receptors on AM. *Ex-vivo*: banana and chocolate increased IL-8 secretion by NHBE.	The flavoring profile of E-liquids increases bronchial epithelial cell apoptosis, causes alveolar macrophage phagocytic dysfunction, and alters airway cytokines.
Zhang et al. (47)	To investigate the cytotoxicity of JUUL e-liquids	Assessment of viability and intracellular Ca^2+^ levels of cultured A549 and Calu-3 airway epithelia exposed to JUUL e-liquids.	Significant cytotoxicity, with the “Mint” flavor being the most cytotoxic. Significant elevations in intracellular Ca^2+^, upregulation of IL-6 and early apoptotic marker Annexin V in “Mint” flavor exposed cells.	JUUL e-liquid exposure results in a loss of airway epithelial cell viability, induces pro- inflammatory responses and apoptosis
Rowell et al. (48)	To characterize the effects of e-liquids on cellular Ca^2+^ homeostasis.	*In vitro:* exposure of Calu-3 and HEK-293T cell lines to EC liquids. *Ex-vivo:* exposure of primary human bronchial epithelial cells (pHBE) to EC liquids.	*In vitro:* Acute e-liquid addition elicits cytosolic Ca^2+^ responses in Calu-3 cells. Banana pudding (BP): acutely elevates cytosolic Ca^2+^ in Calu-3 and HEK-293T cell lines; causes endoplasmic reticulum (ER) Ca^2+^ release and store-operated Ca^2+^ entry (SOCE) (notably, up to 24 h-exposure depletes ER Ca^2+^ stores and inhibited SOCE) and protein kinase C phosphorylation in Calu-3 cells. *Ex-vivo:* BP acutely elevates cytosolic Ca^2+^ in pHBE cell lines.	E-liquids may alter Ca^2+^ homeostasis by short- and long-term mechanisms.
**EC BIOLOGICAL EFFECTS** ***IN VIVO***				
Ghosh et al. (49)	To assess of chronic vaping on pulmonary epithelia.	Proteomic investigation of bronchial brush biopsies and lavage samples from healthy NS, cigarette smokers, and EC users (vapers).	Epithelial cells from biopsy samples revealed ~300 proteins differentially expressed in smoker and vaper airways, with 113 proteins uniquely altered in vapers.	Chronic vaping exerts marked biological effects on the lung.
Staudt et al. (50)	To assess whether acute exposure to EC aerosols modified the biology of the small airways epithelium (SAE), and alveolar macrophages (AM).	Assessment of healthy naïve individuals, with plasma levels of endothelial microparticles (EMP), and bronchoscopy to obtain SAE and AM, at baseline and 1 week later, after the inhalation of EC (*n* = 7 randomized to EC with nicotine and *n* = 3 to EC without nicotine).	Altered transcriptomes of SAE and AM for all subjects and elevated plasma EMP levels following inhalation of EC with nicotine.	Inhalation of EC aerosols dysregulates human lung homeostasis in healthy naïve individuals.

Differential effects in airway epithelial cells from non-smokers and smokers were observed in a recent study comparing mucin response to EC generated aerosols from PG and glycerol (GLY). In details, the aerosol from the GLY exposure increased MUC5AC levels in human nasal epithelial cells (hNECs) from non-smokers. In addition, the PG:GLY with freebase nicotine exposure increased MUC5AC and MUC5B levels in hNECs from non-smokers (41). Of note, e-vapor exposure resulted harmful as tobacco smoking in inducing morphologic differences in secretory function and eliciting acute decline of ciliary beat frequency in airway epithelial cultures of non-smokers (42). In line with these findings, short-term exposure to PG/VG was found to decrease glucose transport in human airway epithelial cells, suggesting that these EC constituents may alter defensive properties of the respiratory epithelium (43). Conversely, Herr et al., when comparing EC-vapor and traditional cigarettes exposure, reported an overall less acute effect of EC on airway-epithelial cell biology, although data *in vitro* did not allow definitive conclusions about EC long-term safety (44).

The comprehensive harmful effect of EC constituents, including E-liquid flavors, nicotine, VG and PG, has been also investigated by Ween et al., showing increased bronchial epithelial apoptosis and macrophage efferocytosis dysfunction in bronchial epithelial cells. Interestingly, the authors found that EC can alter bronchial epithelial cell cytokine secretion pathways in a flavor-dependent manner (45). In a more recent study, the flavoring profile of E-liquids has been found to cause bronchial epithelial cell apoptosis, alveolar macrophage phagocytic dysfunction, and to alter airway cytokines, pointing out the issue of regulating flavored E-liquids and the need of more rigorous studies providing clear data on the health impacts of flavored ECs (46). With particular regard to E-liquids mediated effects, two recent studies reported inappropriate activation Ca^2+^ signaling pathways which in turn can have a detrimental impact on cell function and survival resulting in a loss of airway epithelial cell viability, and also in inducing pro-inflammatory responses and apoptosis (47, 48).

*In vivo* models confirmed that vaping exerts marked biological effects on the lung, as demonstrated by proteomic investigation that opened new research questions to be further explored about the health impact of EC exposure (49). Inhalation of EC aerosols has been found to dysregulate lung homeostasis even in healthy naïve individuals (50). Overall, the available evidence suggests that EC cannot be considered less dangerous than cigarette smoking and that more studies are required to determine which components of EC aerosol and patterns of use contribute to the damage to airway biology.

## Parental Attitudes Toward EC Use and Perception of Children's Health Risks Connected to EC Exposure

The rapid growth in EC use is linked to advertising and promotion marketing campaigns (51, 52) which have likely contributed to increasing favorable perceptions, even among pregnant women. Indeed, EC are thought to be a safer alternative to conventional cigarettes and to help women quit smoking in pregnancy (53), although it has not been demonstrated that using them is effective with regard to the latter (54). Women may be attracted to the flavorings due to alterations in taste that they generally experience during pregnancy (55). Hence, flavors may increase the appeal of EC in this population. In a study conducted by Dobbs et al. on a sample of 219 pregnant women between 18 and 45 years, participants perceived that vaping during pregnancy was significantly less risky than smoking conventional cigarettes (56). A cross-sectional study on 176 pregnant smokers (38% dual users), reported that, among dual users, 41% used their EC daily. Overall, whereas the majority of participants perceived EC use among women as acceptable, far fewer reported this behavior as acceptable during pregnancy. Of note, 56% perceived EC as harmful to women and 53% believed that EC risk harming the fetus (57). Data from the Population Assessment of Tobacco and Health (PATH) study showed that 4.9% of pregnant respondents currently used EC, and most of them concurrently used CC (58, 59). In a more recent study on 69,508 pregnant women from 38 states in the U.S., the weighted prevalence of EC use during the last 3 months of pregnancy was 1.1%. Among women who smoked EC before being pregnant, 24.4% continued to use EC during pregnancy. Among those who smoked EC during pregnancy, 62.3% were dual users. Comparing smokers with non-smokers before pregnancy the AOR was 6.7 (95% CI 4.4–10.3) for EC use during pregnancy (60).

A study on parents in socio-economically disadvantaged circumstances reported that several of them who had tried, currently used, or were planning to use EC considered these products as potentially helpful to protect their children from SHS exposure in the home (7). Notably, some differences in smoke-free habits between parents who vape and parents who smoke tobacco cigarettes have been noticed by Drehmer et al. (61). Smoking parents are more likely to have stricter smoke-free habits in cars and home than EC users or double smokers, who more often allow others to vape when a child is present. Moreover, limited knowledge about the harm of chemical components of e-liquids often leads parents to refill their device in the presence of their children without the necessary safety precautions (61).

It should also be pointed out that parents actively shape adolescent behavior with regard to EC use (62). Social factors including peer or familial attitudes have a great influence on susceptibility to becoming smokers or vapers. Youth with vaping parents are significantly more likely to start vaping than others (63). The determinants of this association may be emulation of parental habits and low awareness of parental disapproval among children and adolescents. Generally, a higher frequency of EC use has been described among fathers, but the role of maternal use in inducing adolescent vaping seems to be more influential (1). Parents who smoke or vape, especially mothers, are more tolerant toward their children vaping than non-smoking/vaper mothers. A positive association between maternal and adolescence vaping was shown by Sabbagh et al. (1). Patel et al. demonstrated that a significant percentage of parents of middle- and high-school children have poor knowledge about the commercial ENDS variety and significantly underestimates their own children's vaping risk. The likelihood of their intervention in preventing or stopping vaping-risk behaviors is dramatically low (64).

Overall, these findings suggest a low parental perception of children's health risks connected to EC exposure during both prenatal and post-natal life.

A pediatric-office based intervention may be a successful strategy to advise parents about the risk connected to EC exposure for their children's health. A recently published policy statement of the American Academy of Pediatrics recommends that pediatricians screen for EC use and exposure in clinical practice; provide counseling that homes, cars, and places where children live should have comprehensive tobacco-free bans that include EC; do not recommend EC as a tool for smoking cessation (65). Based on the established relationship of trust with parents, pediatricians could have a vital role in prevention of EC use. Indeed, the pediatric visit may be an opportunity for pediatricians to offer smoking-cessation treatment to parents and caregivers because parents generally see their children's pediatrician more often than their own healthcare provider. Pediatricians can identify children exposed to passive smoke and help parents and caregivers to quit, connecting them to state quit-lines or to local cessation services, or prescribing them with nicotine replacement therapy (NRT) (66). Even brief advice can increase smoking cessation rates (67). The Clinical Effort Against Secondhand Smoke Exposure (CEASE) is a program for addressing parental tobacco dependence that can be easily implemented in the pediatrician's office. CEASE focuses on the principles of ask, assist, and refer. In a 2-year randomized clinical trial, implementing this program within pediatric offices resulted in significantly higher rates of tobacco treatment delivery, like prescription of NRT or quit line enrollment, and a decline in parental smoking rates in comparison with usual care (2.7 vs. 1.1%, respectively). This latter finding was objectively confirmed by measurements of salivary cotinine in parents, which demonstrated that smoking cessation can be achieved, if pediatricians routinely screen parents for tobacco use and offer smoking cessation treatment (68). In spite of this evidence, pediatricians reported poor self-efficacy about smoking cessation screening and counseling of parents and youth. Overall, they were more confident about counseling youth than parents (*p* < 0.01) and they identified pregnancy as the preferred time window for their intervention. Moreover, though 93% considered EC as dangerous as CC, 34% never counseled youth about the hazards of vaping (69). This finding provides evidence that an adequate level of awareness is required among pediatricians to improve communicative skills and strategies that could change the parental approach to vaping.

Objective measurements of EC exposure in children might help pediatricians to increase parental perception of children's health risks connected to EC exposure. Preliminary findings of a study conducted on cigarette-exclusive, EC-exclusive, and non-users and their children showed that caregivers using EC perceived them as less harmful, and reported using them more frequently at home and in the car, even when their children were present, compared to cigarette users. Indeed, no significant difference in levels of urinary cotinine was found between children from the cigarette user group and those from the EC user group, that appeared to be exposed to nicotine at levels similar to children living with cigarette users (70). These findings are in accordance with previous results obtained in adults (71) and underline the need for targeted educational interventions for caregivers around the potential harms of EC exposure in children. Similar interventions should also involve adolescents, as a recent study reported significantly greater toxicant exposure in EC users compared with non-using peers. In particular, EC–only users had up to 3 times urinary levels of 5 Volatile Organic Compounds (VOCs). Since many of the VOCs were carcinogenic, and they were present whether the product contained nicotine or flavorings, EC users must be aware of the potential risk from being exposed to carcinogenic compounds generated by these products (72).

In summary, the involvement of pediatricians, nurses, and other pediatric health care professionals is highly valuable. Given the high prevalence of mental health problems in smokers, EC users and drug users, pediatricians, and pediatric health care professionals should carefully address all smoking and vaping related health and mental health issues concurrently. These issues often cannot be separated or addressed in isolation without working on all the others in order to deliverer effective patient care.

## EC Use and Exposure During the COVID-19 Pandemic

With the implementation of social distancing due to Coronavirus Disease 2019 (COVID-19) pandemic, isolation from the school environment, loneliness, stress, and poor social support could contribute to an increased EC use, particularly in adolescents with pre-existing mental health conditions (73). In addition, social media platforms for EC marketing, commonly frequented by adolescents, have spread several unfounded health claims about a protective role of EC against COVID-19 (74). Therefore, it is not surprising that concerns have been recently raised about the association of vaping, and its related effects, with COVID-19, mainly in youth (75). Indeed, exposure to toxicant compounds in EC suppresses immune function, increasing susceptibility to infections (76). This explains why EC and dual users show a higher risk of COVID-19 diagnosis in comparison with non-users. Moreover, similar to smokers, it is plausible that vapers could experience worse health outcomes with respect to non-smokers. In particular, COVID-19 may cause acute respiratory failure and ground glass opacities on chest imaging, that may overlap with clinical manifestations and radiologic findings of EVALI. As a further complication, the psychological consequences of the COVID-19 pandemic may contribute to increased cases of EVALI, given that EVALI patients reported vaping more to cope with pandemic associated stressors and anxiety (75–77). Moreover, EVALI and COVID-19 may co-exist in some cases. Indeed, a significant number of EVALI patients reported sharing the same device with friends and family members, increasing their risk for SARS-CoV-2 infection (78). Both EVALI and COVID-19 share some clinical features like fever, cough, respiratory distress and non-specific gastrointestinal symptoms. An elevation of inflammatory markers is observed in both conditions, as well as radiological findings like bilateral multifocal ground glass opacities, with or without consolidation on chest CT imaging. However, EVALI remains an exclusion diagnosis. Therefore, eliciting any vaping history in adolescents with unexplained respiratory distress is crucial in making the diagnosis, along with testing for SARS-coV-2 infection. Indeed, treatment of EVALI differs from COVID-19, and early initiation of steroids can be lifesaving and may reduce the length of hospitalization (79).

Further studies addressing the relationship between EC use and exposure and COVID-19 are required. Though youth are generally considered at lower risk of developing COVID-19 than older individuals, efforts should be made in order to reduce youth vaping and identify those susceptible to severe disease outcomes (80). Longitudinal data are needed to explore whether the detrimental interplay between EC use and COVID-19 might exacerbate lung injury in children affected by underlying conditions, such as asthma. Further studies are also required to investigate the long-term impact of SARS-CoV-2 infection on respiratory health of children with or without underlying lung diseases exposed to EC.

## Conclusions

The widespread use of EC is an emerging threat to children's health. Studies have provided evidence about the health risks of EC passive exposure, which are, in some respects, comparable to those associated with passive tobacco exposure. However, some research gaps should be addressed. First of all, the risks of smoking EC during pregnancy need to be thoroughly elucidated; in particular, further studies are needed to evaluate the placental vulnerability to EC exposure. Secondly, prenatal effects of the chemical components of e-vapors have not been thoroughly defined in human studies so far. Research gaps also include lack of generalizability and adjustment for confounders like tobacco use, given that most of the subjects enrolled in the aforementioned studies are dual users.

Little information is available regarding the effect of EC exposure on children's respiratory health. Available data are mainly self-reported and may be subjected to recall bias. Therefore, findings should be corroborated with medical records, clinical assessment measures, or examination by physicians. Moreover, the long-term impact of EC smoking on respiratory symptoms and pulmonary function needs to be further elucidated, especially in patients with EC-associated lung injury. Last but not least, more studies are required to determine which components of EC aerosol and patterns of use contribute to the damage to airway biology.

In the context of the current COVID-19 pandemic, longitudinal data would be helpful to explore whether the detrimental interplay between EC use and COVID-19 might exacerbate lung injury in children affected by underlying conditions, such as asthma. Further studies are also required to investigate the long-term impact of SARS-CoV-2 infection on respiratory health of children with or without underlying lung diseases exposed to EC.

The low parental perception of the risks connected to EC exposure for children throughout different vulnerability windows increases their susceptibility to harmful effects from passive vaping, including prenatal damage and post-natal respiratory effects, and increases the risk of vaping among adolescents. Since the child's home is the primary source of exposure to tobacco smoke, addressing the issue of parental perception of the harmful effects of EC exposure in children is essential for promoting health, especially in more vulnerable children such as those with respiratory chronic diseases like asthma.

Pediatricians could play a crucial role in increasing parental perception of children's health risks connected to EC use and exposure; at this purpose, they would benefit from receiving *ad-hoc* training courses for promoting smoking cessation in parents and adolescents (81). In this context, further studies should explore the involvement of pediatricians in smoking cessation programs for parents and adolescents.

## Author Contributions

FM and SL: conceptualization. FM and GF: writing original draft. GF and SL: review and editing. All the authors read and approved the final version of the manuscript.

## Conflict of Interest

The authors declare that the research was conducted in the absence of any commercial or financial relationships that could be construed as a potential conflict of interest.

## Publisher's Note

All claims expressed in this article are solely those of the authors and do not necessarily represent those of their affiliated organizations, or those of the publisher, the editors and the reviewers. Any product that may be evaluated in this article, or claim that may be made by its manufacturer, is not guaranteed or endorsed by the publisher.

## Abbreviations

AOR: adjusted odd ratio
DLCO: diffusion capacity of carbon monoxide
EC: E-Cigarettes
ENDS: electronic nicotine delivery system
EVALI: EC vaping associated lung injury
GLY: glycerol
RR: relative risk
PG: propylene glycol
SGA: small-for-gestational-age
SHS: second-hand smoking
VG: vegetable glycerin.
